# MAESTROS: A Multiwavelength Time-Domain NIRS System to Monitor Changes in Oxygenation and Oxidation State of Cytochrome-C-Oxidase

**DOI:** 10.1109/JSTQE.2018.2833205

**Published:** 2018-05-09

**Authors:** Frédéric Lange, Luke Dunne, Lucy Hale, Ilias Tachtsidis

**Affiliations:** 1 Biomedical Optics Research Laboratory Department of Medical Physics and Biomedical Engineering University College London London WC1E 6BT U.K; 2 Biomedical Optics Research Laboratory Department of Medical Physics and Biomedical Engineering University College London London WC1E 6BT U.K; 3 Electronic and Electrical Engineering University College London London WC1E 7JE U.K

**Keywords:** Time domain measurements, spectroscopy, biomedical engineering, laser biomedical applications, photomultipliers, scattering

## Abstract

We present a multiwavelength, multichannel, time-domain near-infrared spectroscopy system named MAESTROS. This instrument can measure absorption and scattering coefficients and can quantify the concentrations of oxy- and deoxy-haemoglobin ([HbO_2_], [HHb]), and oxidation state of cytochrome-c-oxidase ([oxCCO]). This system is composed of a supercontinuum laser source coupled with two acousto-optic tuneable filters. The light is collected by four photomultipliers tubes, connected to a router to redirect the signal to a single time-correlated single-photon counting card. The interface between the system and the tissue is based on optical fibres. This arrangement allows us to resolve up to 16 wavelengths, within the range of 650–900 nm, at a sampling rate compatible with the physiology (from 0.5 to 2 Hz). In this paper, we describe the system and assess its performance based on two specifically designed protocols for photon migration instruments, the basic instrument protocol and nEUROPt protocols, and on a well characterized liquid phantom based on Intralipid and water. Then, the ability to resolve [HbO_2_ ], [HHb], and [oxCCO] is demonstrated on a homogeneous liquid phantom, based on blood for [HbO_2_], [HHb], and yeast for [oxCCO]. In the future, the system could be used to monitor brain tissue physiology.

## I. Introduction

Near-infrared spectroscopy (NIRS) is now a common tool to monitor oxygenation levels in the brain [1], [2]. The basic principal of NIRS is to measure the change in attenuation of the light between an injection and a collection point separated by few centimetres [3]. The attenuation of the light is due to the different optical properties of absorption and scattering of the constituents of tissue [4]. In the typical optical range used in NIRS (650 to 900 nm), the main absorbers are the water and haemoglobin. Haemoglobin is the protein within the red blood cells that binds and carries the oxygen through the body [5]. It can either be bound to oxygen, oxy-haemoglobin (HbO_2_), or not bound to oxygen, deoxy-haemoglobin (HHb). NIRS is able to quantify the concentrations of these two chromophores because they have well distinguished optical spectra [6]. From these concentration measurements, useful clinical information about the tissue oxygen status can be extracted, like tissue saturation (SaO_2_ = HbO_2_/(HHb + HbO_2_)) [7]. The majority of NIRS systems are based on continuous wave (CW) technologies, that measure changes in light attenuation at a small number of discrete wavelengths (typically two or three) [8]. CW means here that only changes in the intensity of light due to the attenuation by the tissue is considered. Then, by assuming that the scattering and water concentration do not change, the changes in light attenuation are linked to the variation of the light absorption due to the changing concentrations of HbO_2_ and HHb. Indeed, the changes in attenuation are linearly linked to the change in chromophore concentrations as modelled by the modified Beer-Lambert law [9]. 

In addition to these two chromophores, NIRS is able to detect a third chromophore, cytochrome-c-oxidase (CCO) [10]. CCO is the terminal electron acceptor of the electron transport chain in the mitochondria which is responsible for 95% of cellular oxygen metabolism. NIRS monitors the changes in the redox state of CCO using the oxidised minus reduced CCO spectra to obtain a measurement of changes in the concentration of oxidised CCO ([oxCCO]). Thus, by coupling the oxygenation information, obtained with the haemoglobin measurement, and the CCO measurement, which is a key indicator of oxidative metabolism, NIRS can provide a clearer picture of the tissue function and/or health [11]. However, even though monitoring [oxCCO] was first suggested in 1977 by Jöbsis [12], it has not been exploited widely in the NIRS community. This is explained by the higher complexity of the instrumentation needed to extract that third chromophore. Indeed, there are two main challenges to overcome to be able to measure it. Firstly, the concentration of oxCCO is lower than that of haemoglobin by about one order of magnitude [10]. Secondly, the oxCCO contrast is dominated by a broad absorption peak of the CuA center around 820–850 nm. These challenges (low concentration and lack of easily identifiable optical signature) make the quantification of oxCCO difficult, often leading to crosstalk effects with haemoglobin [13]. Broadband NIRS, that uses over a hundred wavelengths, has successfully proven capable in tackling these two issues and accurately resolving the changes in concentrations of [HbO_2_ ], [HHb] and [oxCCO] without crosstalk. For example, it has been shown that broadband NIRS can quantify these 3 chromophores accurately during functional activation in adults  [14]–[18] and babies [19] ; in brain injury in babies [11], [20] , and during some physiological challenges on animals [21] and humans [22]. The interested reader can refer to the recent review of Bale and colleagues [10] for more details.

Even though the use of broadband NIRS gives access to information about metabolism, the use of CW techniques still suffers from intrinsic limitations. First of all, the CW measurement is based on the assumption that the change in light attenuation is due to a change in the absorption coefficient only [8]. However, light attenuation in tissue is related to both the absorption and scattering coefficients. Thus, by not measuring the scattering information, classical CW systems cannot deliver absolute values of chromophore concentrations and provide only a change in chromophore concentrations from an unknown baseline. It can also lead to the wrong scaling of the concentration changes due to crosstalk between absorption and scattering, and lack of the optical pathlength measurement. Indeed, to solve the modified Beer-Lambert equation, one needs to input a scaling factor called the differential pathlength factor (DPF) [23]. This parameter accounts for the longer path of the light between the source and the detector due to the high scattering in tissue. Thus, the DPF used is often taken from tabular values [24]. Secondly, the penetration depth of CW instruments is limited [25]. In the case of brain measurements, light needs to pass through the skin, the skull and the cerebrospinal fluid (CSF) - collectively referred to as the extra cerebral layer (ECL) - before reaching the brain. In adults, the ECL depth has been measured between 10 and 30 mm [26]. Thus, the limited penetration depth of CW systems affects the origin of the measured contrast (i.e., ECL or brain?). However, CW instruments can resolve this issue by employing a multi-distance approach, which considers the penetration depths of photons as a function of the source detector separation distance [27]. By using a short source detector separation and a long separation, one can estimate the variation in the chromophores’ concentrations of the shallow and deep tissues respectively.

Another NIRS technique, called time resolved (TR) or time domain (TD) NIRS, can overcome these limitations [28], [29]. TD NIRS is a technique that measures the time of flight of photons. It uses ultra-short pulsed light sources, typically laser pulses of femto or picoseconds, and measures the time point spread function (TPSF) of the light after it has travelled through the tissue. The initial pulse gets attenuated, due to the absorption, and broadens, due to the scattering. Thus, the information of the distribution of the arrival time of photons gives the possibility of extracting absolute absorption and scattering coefficients, leading to an absolute measurement of chromophore concentrations [30]. The other possibility offered by TR systems is to recover depth information, by separating the early from late photons, corresponding to superficial and deep tissue. Indeed, the longer the arrival time of the photon, the higher the probability that the photon has probed deep tissue. Hence it provides a better knowledge of the origin of the contrast measured, whether ECL or brain [31], [32].

That extra information comes at the cost of the complexity and size of the system used. However, since the first development of TD systems, based on STREAK cameras [30], a lot of developments in photonic devices have allowed simplification of the instrumentation and better acceptance of the technology. The development of the time-correlated single-photon counting (TCSPC) technique [33], allowed a reduction in the size of the systems, as well as improving the dynamic range, robustness and sensitivity, down to the single photon level detection. Moreover, the recent development of compact pulsed laser sources based on supercontinuum generation permits the development of true time-resolved spectroscopic systems, with a higher versatility in wavelength selection and the possibility of using more than just two or three wavelengths. Several TD systems based on supercontinuum sources have been developed. These systems use different techniques either based on the use of an imaging spectrometer coupled with ICCD [34], [35] or streak cameras [36], or based on the use of photo-detectors like silicon photomultiplier (SiPM) or photomultiplier tubes (PMT) coupled with TCSPC cards [37]. By temporally multiplexing the wavelength, one can also use several pulsed laser diodes as sources [38]. The selection of a particular acquisition scheme is often dictated by the targeted application, since a trade-off has to be made between the field of view (mean area covered depending on the source/detector numbers), the acquisition frequency, the number of wavelengths and the SNR. For example the broadband system developed by Konugolu Venkata Sekar and colleagues [39], intended to measure precisely the optical properties of tissue, covers a bandwidth between 600 and 1350 nm. However, it requires a few minutes to acquire a single measurement of the whole bandwidth, which is much too long to be able to follow the physiology. But it is worth noting that those types of system have access to other chromophores than just [HbO_2_], [HHb] or [oxCCO]. For example, in [39], the tissue composition of [HbO_2_], [HHb], water, collagen and fat is retrieved. It gives a better insight into the tissue composition than just the haemoglobin content, but a trade-off has to be made between the acquisition frequency, and the number of chromophores retrieved.

We intend to develop a system focused on the detection of the dynamic cerebral changes of [HbO_2_], [HHb], and [oxCCO] *in-vivo*, which is a real challenge. Indeed, ideally, it would require the acquisition of the TPSFs of hundreds of wavelengths, in a time scale compatible with physiology (typically 1s). Moreover, multiple sources and/or detectors would be required since the monitoring of more than one location is often useful in order to obtain relevant physiological changes over a specific area (i.e., for example when performing functional brain activation tasks). Hence, matching those specifications seems out of reach.

However, a recent study showed that it was possible to drastically reduce the number of wavelengths needed to retrieve [oxCCO] [40]. Indeed, Arifler and colleagues demonstrated that using only 3 wavelengths to resolve [oxCCO] lead to an error of up to 10%, compared to a gold standard calculation using 121 wavelengths as obtained by broadband NIRS. This error could be reduced to 4% when using 4 or 5 wavelengths and to even less than 2% with 8 wavelengths. Thus, we have developed such an instrument that temporally multiplexes up to 16 wavelengths, with an acquisition frequency able to follow dynamic physiological changes.

Preliminary system description has been provided by Dunne and colleagues [41] , and Lange and colleagues [42]. In the present work, Section II will describe the instrument in depth, both in terms of the hardware and software. Section III will firstly describe a basic characterisation of the system using the BIP [43] and the nEUROPt  [44] protocols, and on a simple liquid phantom. Then, it will describe the capability of the instrument to detect haemoglobin and oxCCO variations using a liquid phantom based on blood, Intralipid and yeast. Lastly, in Section IV, we will discuss the present results together with our previously reported *in-vivo* work reporting the evaluation of the response of haemoglobin and oxCCO in the left arm during the occlusion of the brachial artery.

## II. System Description

### A. Overview

**Fig. 1.**
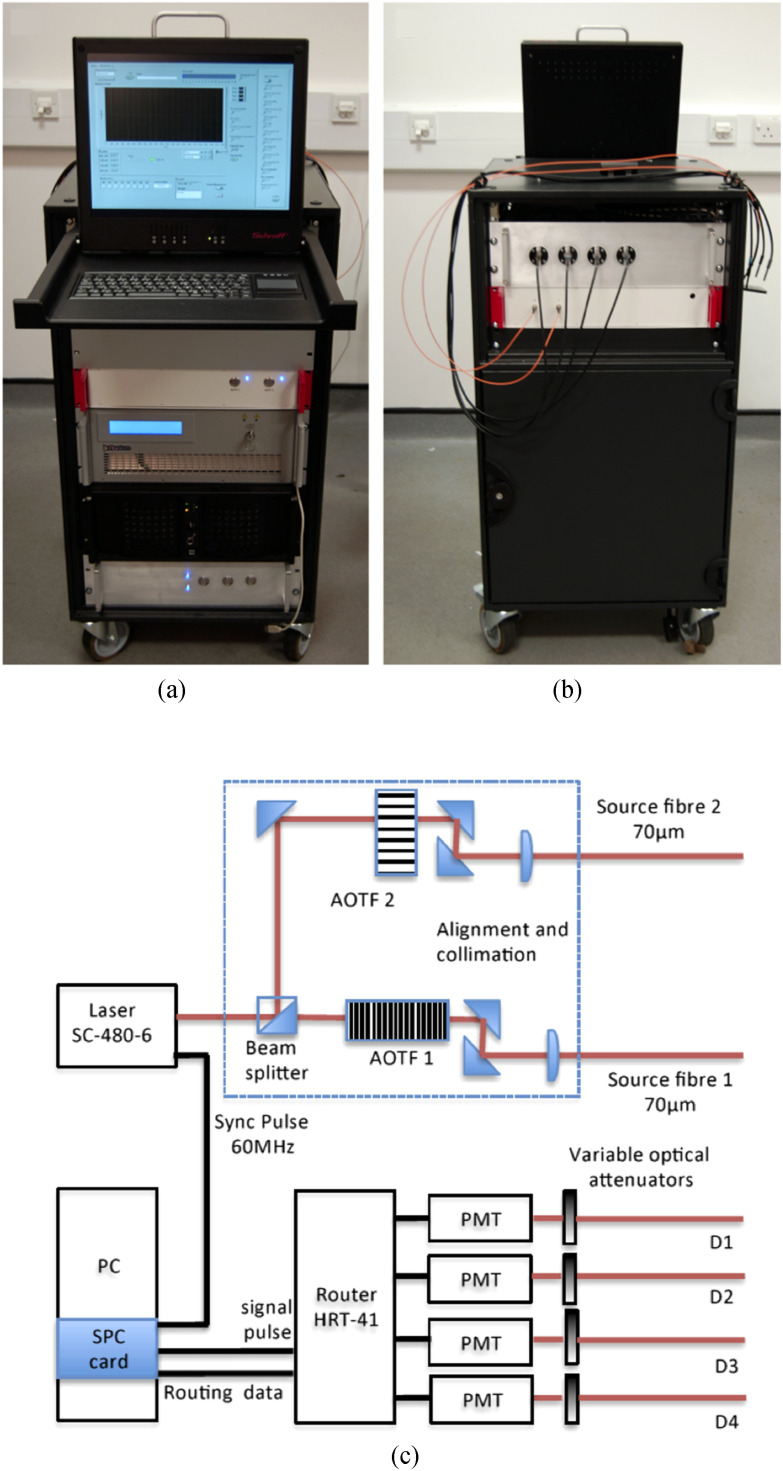
(a) Picture of the front of the system, (b) Picture of the rear of system, showing fibre optic connections. (c) Schematic illustration of the major system components.

Fig. 1 shows the completed system, named MAESTROS (Metabolism and hAemoglobin Evaluation via a Spectroscopic Time Resolved Optical System) and schematic of the components. A supercontinuum laser produces broadband light which passes through a dual acousto-optic tuneable filter (AOTF). The AOTFs allow the wavelength selection in the NIR range. It worth noting that for our application, we are particularly interested in the wavelength range between 780 and 900 nm, which has been proven to be a good wavelength range in order to resolve oxCCO accurately [40]. The two spectrally filtered beams are then injected into two optical fibres that takes the light to the sample (e.g., head). The reflected light is then collected by up to four optical fibres, separated from the source by a few centimetres. The collected light first passes through custom made variable optical attenuators (VOAs), in order to adjust the signal dynamics before reaching the photomultiplier tube (PMT). The four PMT outputs are connected to a four-way router, to redirect the signal to a single time correlated single photon counting card (TCSPC). This optical scheme allows us to measure the four different Temporal Point Spread Functions (TPSF) from each of the four PMTs.

### B. Emission Stage

**Fig. 2.**
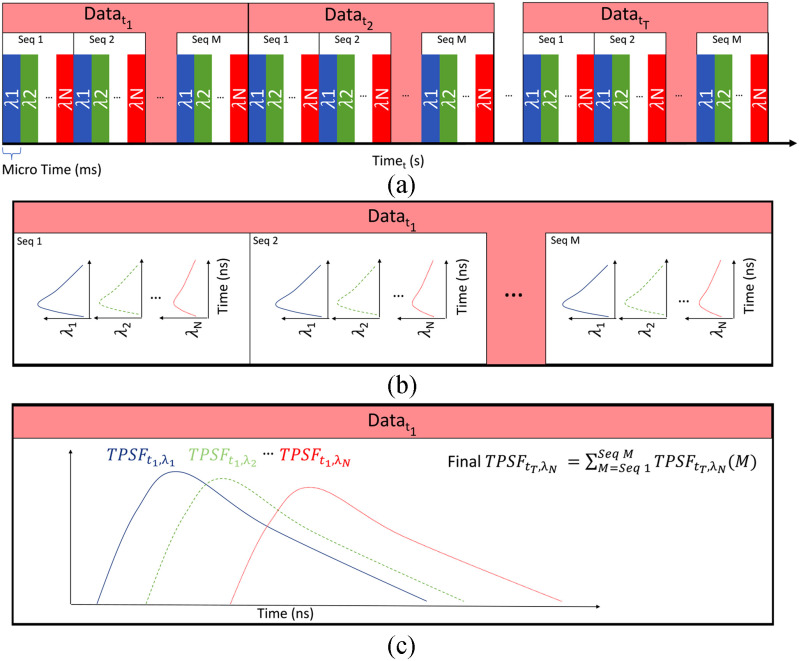
Diagram of the acquisition sequence. (a) Diagram of the timing of the acquisition sequence. The TPSF at each wavelength is acquired sequentially with a small integration time per wavelength (10th of microseconds). Then this sequence is repeated M times. This operation is repeated for each time point. (b) Example of the acquisition of the TPSF for the first time point. This illustrate the sequential acquisition of the TPSF for each wavelength. (c) Final TPSF for the first time point. All the TPSFs of every sequence within the first time point are summed up at each wavelength and saved in the same file.

The laser source is a supercontinuum SC-480-6 (Fianium, UK). It has a total power of 6 W and a spectral bandwidth ranging from 400 to 2100 nm. The laser produces pulses of 4 picoseconds full-width half maximum (FWHM) at a fixed repetition rate of 60 MHz. The output beam of the laser is then directed via a polarising beam splitter to two modified Gooch and Housego acousto-optic modulators (2986-01). The standard FWHM of AOTF crystals is 5 nm, however, we are using a modified crystal to achieve a manufactured stated FWHM of 2-3 nm in the NIR range. Moreover, these AOTF provides a special fast switching mode, permitting the switching of 16 wavelengths between 650 and 1100 nm at 160 Hz. Typically, the signal at each wavelength is acquired sequentially for within a micro time (MT) of 20 to 50 ms. Then, the measurement is repeated several times, and the signal acquired for each MT and each wavelength is summed to achieve a good photon count. Thus, it leads to an acquisition frequency ranging from 0.5 to 2 Hz, depending on the number of wavelength used, the number of repeats and the MT. A diagram of this acquisition sequence is shown in Fig. 2.

**Fig. 3.**
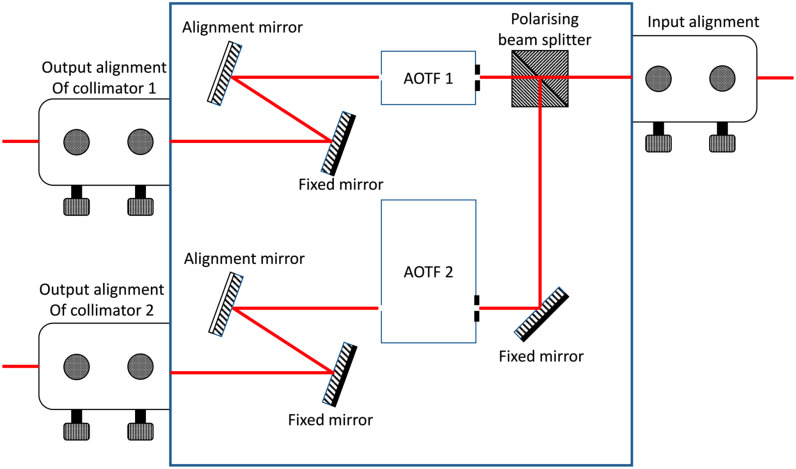
Optical arrangement of the AOTF. The input beam is first split by a beam splitter and directed to two separate AOTFs. Then the beam at the outputs of the AOTFs are focused into two separate optical fibres.

After passing through the two crystals, the two spectrally filtered beams are then injected into two, 2 m long optical fibres (GI 70 µm core, NA: 0.54, Loptek), that carries the light to the sample. Fig. 3 shows a schematic of the two AOTFs arrangement.

### C. Detection Stage

**Fig. 4.**
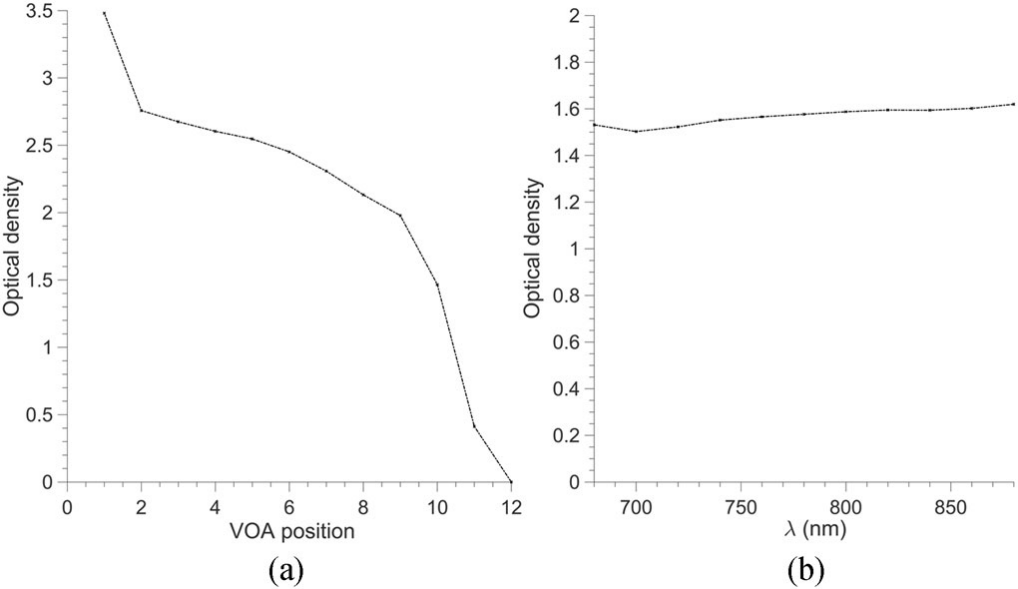
Optical properties of the VOA. (a) Optical density of the 12 positions of the VOA. (b) Spectral characteristic of position 10.

The diffused light from the sample is then collected by up to four, 2 m long optical fibre bundles (3 mm core, SI fibres, NA: 0.64, Loptek). The collected light first passes through custom made variable optical attenuators (VOAs). The design of those VOAs are based on that of MONSTIR [45] and are composed of X-Ray films that have been exposed with variable exposure time to achieve different optical density (OD) values. Eleven different OD values are created and mounted on a rotating wheel controlled by a stepper motor. Therefore, the range of the OD useable by the system varies from 0 to 3.7 in 12 steps, the first one being left blank (i.e., without any OD). Fig. 4 presents the typical OD values for the 12 steps together with the typical wavelength dependency of the OD values.

Four Hamamatsu H7422P-50 photomultiplier tube modules are used to detect the photons. They are all maintained at a constant temperature of 5 °C by a Peltier cooling element next to the photocathode, in order to reduce thermal noise and dark count.

The output of each PMT is connected to a four-way router (HRT-41, Becker and Hickl), to redirect the signal to a single TCSPC card (SPC-130-EM, Becker and Hickl). This card has a minimum time channel width of 813 fs and a time window resolution of up to 4096 channels. The electrical time resolution is less than 3 ps RMS with a 6.5 ps FWHM which will be negligible compared to the system's instrument response function (IRF) (see Section III-A).

### D. Hardware

All of the elements of the system are mounted into a 19” rack mounted on castors, to make it transportable. The final dimensions of the system are 95 × 60 × 110 cm. A 1000 VA Reo medical isolation transformer which complies with the EN60601 standard (3rd edition) is used to achieve a low general earth leakage current to protect the system.

### E. Software

**Fig. 5.**
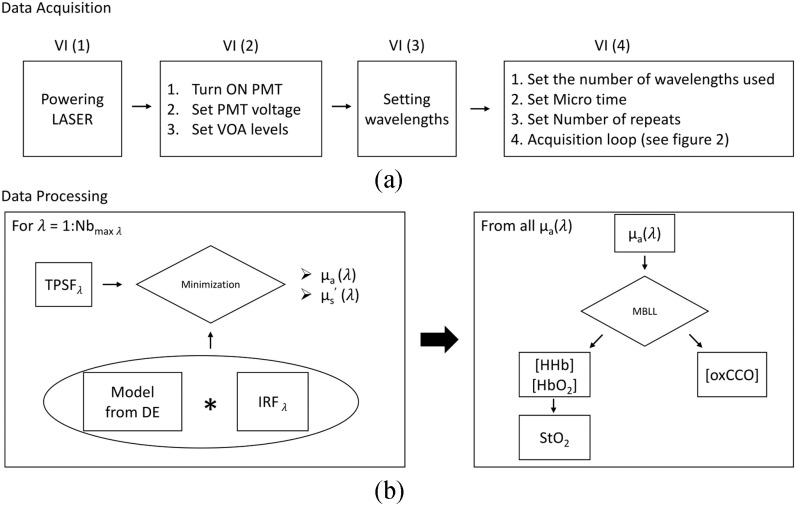
(a) Flowchart of the acquisition software. VI: Virtual instrument. (b) Typical flowchart of the data processing steps. DE: Diffusion Equation, IRF: Instrument Response Function. MBLL: Modified Beer-Lambert Law.

The software controlling the system is written in LabVIEW (National Instruments). Separate user friendly VIs are used to control: (1) the laser power, (2) PMTs voltage and cooling and VOA levels, (3) the AOTF wavelengths used and (4) data collection, by setting the parameters of the TCSPC card and the fast wavelength switching. Simplified flowcharts of both the software and the typical data processing steps is provided in Fig. 5. The data processing workflow is detailed in Section III-D.3.

## III. Performance Assessment

In this section, we will describe the performance assessments of the system. We have firstly assessed the performances of our system using two well established protocols, namely the BIP protocol [43], for the very basic assessment of the component; and the nEUROPt protocol [44], for the basic assessment of the capabilities of the system to detect an absorption change in depth. We also used a 1% aqueous solution of Intralipid to assess the accuracy of the optical properties retrieved by our system. Finally, we have developed a liquid phantom to specifically assess the capability of the system to detect haemoglobin and oxCCO. We performed a full characterisation of the system (all sources, detectors, and wavelength), which has produced a very large amount of data. For clarity reasons, we will present here a subset of data representative of the system performance. We will specify each time which detector, source, and wavelength was used.

### A. Basic Instrument Protocol (BIP)

As we intend to perform a spectroscopic analysis, both the power and the spectra quality, in terms of resolution (FWHM) need to be carefully assessed. First of all, the power need to be high enough over the range that we are targeting (780 to 900 nm). Typically, time resolved systems reported in the literature have a power of around 1 mW per wavelength [46]. Thus, we have targeted this value to ensure a good SNR for our measurement. Secondly, the resolution of a spectroscopic system is a key parameter, in our case dictated by the bandwidth of each wavelength selected by our AOTF, in order to retrieve accurate results. Indeed, it has been shown that a large bandwidth can lead to spectral distortion in the recovered spectra [47].

**Fig. 6.**
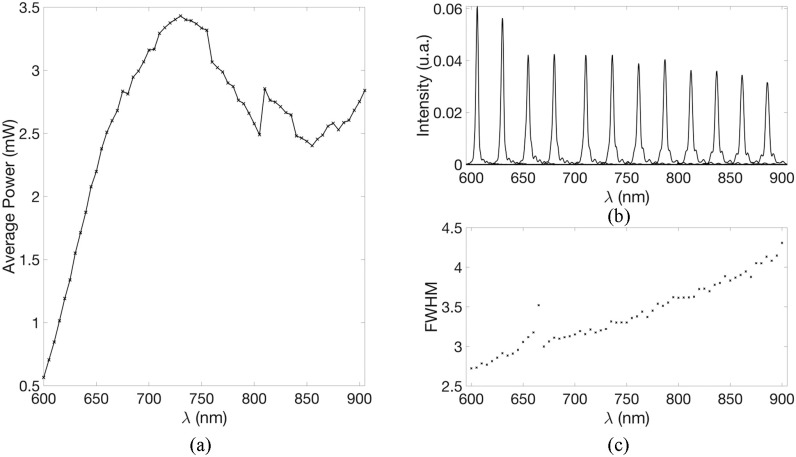
(a) Typical power (b) spectra and (c) FWHM of the source between 600 and 900 nm, in steps of 5 nm.

In Fig. 6(a) we report the typical maximum output power of the source, measured using a photodiode (PMD100, Thorlabs), from 600 to 900 in steps of 5 nm, while Fig. 6(b) and (c) report the spectral shape and FWHM of those wavelengths respectively. In Fig. 6(b) we report only few wavelengths for clarity.

The maximum power does not exceed 3.4 mW (at 730 nm), This power is sufficient for our application. Moreover, according to the British Standard BS EN 60825-1: 2014, accounting for the physical size and numerical aperture of the source fibres and the operating wavelengths, the maximum permissible exposure (MPE) for MAESTROS to remain skin and eye safe in all circumstances is ${\text{3}}\,\times
       \,{\text{10}}^{- 7}\,{\text{J m}}^{- 2}$ per laser pulse. We then can consider the system as eye safe since the exposure for a power of 3.4 mW is of ${\text{1.2}}\,\times \,{\text{10}}^{-
       7}\,{\text{J m}}^{- 2}$ per laser pulse. Furthermore, the output power can be reduced, if needed, via the software. Finally, the 1 mW lower threshold is obtained from 620 nm onwards, which is well below our targeted bandwidth. Regarding the spectral feature, we see in  Fig. 6(c) that the AOTF provides narrow peaks with a FWHM ranging from 2.7 nm (at 600 nm) to 4.3 nm (at 900 nm). These are comparable to other instruments such as the 5 nm reported for MONSTIR II [48]; the 3 nm (at 600 nm) and 7.5 nm (at 1350 nm) reported for the clinical broadband instrument of Politecnico di Milano [39]; and the 3.4 nm (at 826 nm) and 5.2 nm (at 687 nm) reported for the compact multi-channel instrument of Politecnico di Milano [49]).

**Fig. 7.**
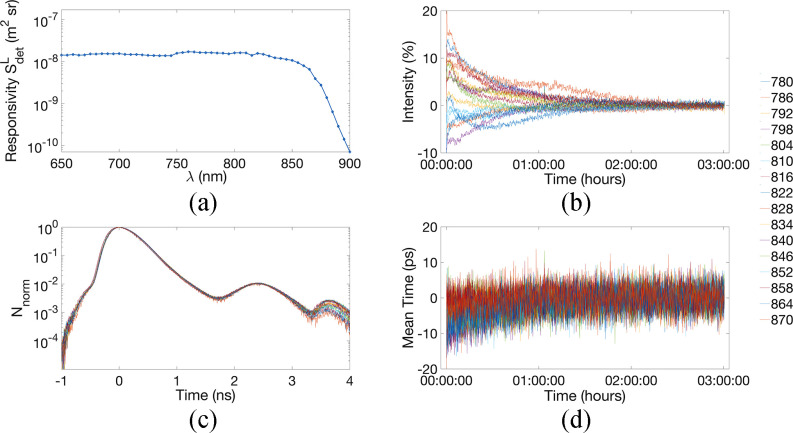
Summary of the basic characteristics of the system. (a) Responsivity of detector 2 (b) Stability of the intensity of the IRF for 16 wavelengths. Intensity expressed as the percentage of variation regarding the mean value of the last 30 minutes. (c) IRF of 16 wavelengths of detector 2 (d) Stability of the mean time of flight of the IRF for 16 wavelengths. Mean time expressed as the mean time of flight variation regarding the mean value of the last 30 minutes.

The typical responsivity of our detection scheme is illustrated in Fig. 7(a), with the responsivity for detector 2, over the range 650 to 900 nm, in steps of 5 nm. If we compare that result to previous reported results in the literature, notably with the one initially reported in the BIP protocol [43] description study, our system is in the range of the others developed in [50], or [51], (ranging between ${\text{1e}}^{- 8}$ and ${\text{1e}}^{-
      7}\,{\text{m}}^{2}{\text{sr}}$), and above MONSTIRII [48] developed at UCL, which is around ${\text{3e}}^{-
      9}\,{\text{m}}^{2}{\text{sr}}$. Moreover, the responsivity of the system is flat over a large bandwidth, between 650 and 850 nm, as expected from the sensitivity of our PMT.

The IRF presents a FWHM of 465 ps at 800 nm for detector 2. The IRF of the other wavelengths present similar characteristics, as seen in Fig. 7(c) that shows the IRF for detector 2 at the 16 wavelengths used for the phantom experiment reported in Section D (from 780 to 870 in steps of 6 nm), after subtraction of a constant background and the normalization to its maxima and shifted to peak at t = 0. For these 16 wavelengths the mean FWHM is 459+/−9.5 ps. The other detectors present the same characteristics. We also note the typical after peak due to the internal reflection in the system. This after peak is happening at long arrival times and its magnitude is lower than 1% of the main peak. Regarding the afterpulsing, we have calculated the value of the afterpulsing ratio (i.e., according to equation (7) in [43]) to be 0.0015.

Finally, Fig. 7(b) and (d) demonstrate the stability of the IRF for these 16 wavelengths, in terms of photon counts and mean time of flight (MTOF). The stability is defined as +/−1% of intensity variation, and +/−5 ps of mean time of flight variation, regarding the last 30 minutes of the recording. We see that the system requires between 60 and 120 min, depending on wavelengths to reach its stability, which is in the range of previously developed TR systems [43], [49].

### B. Estimation of the Optical Properties of a Liquid Solution

In order to assess the ability of our system to retrieve accurate optical properties over all of our four detectors, we have measured the optical properties, for 16 wavelengths between 650 and 875 nm in steps of 15 nm, of a 1% aqueous solution of Intralipid. The solution was prepared and poured into a dedicated cell to perform the measurement [52]. It consists of a black cell (120 mm × 140 mm × 50 mm) with a series of 5 transparent windows on the front and rear walls. The measurements for the 4 detectors were made in series. The source was positioned and left at the same position for all the measurement series. A window located at 2 cm from the source was used. The measurement was done on the same solution and all four detectors were positioned in series at the same location. The IRF for all the wavelengths and all the detectors was acquired at the end of the measurement series. This way we can look at the typical spectral shape of the absorption spectra of the water within the spectral range covered by our system.

**Fig. 8.**
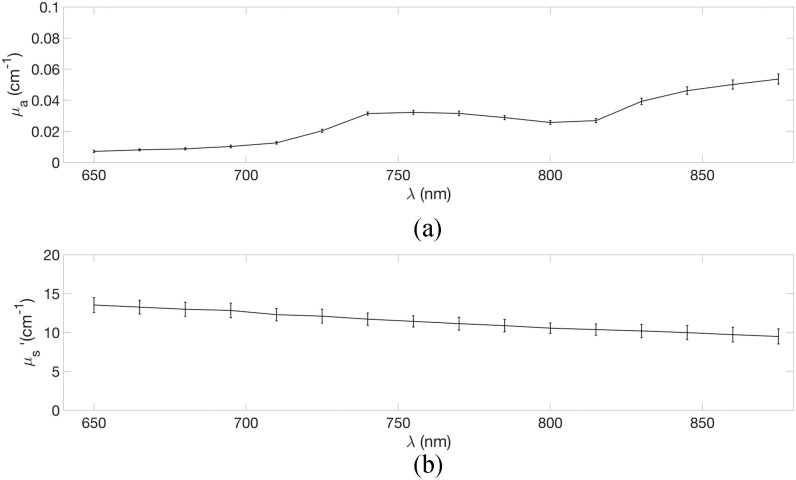
(a) Absorption spectrum of water as measured in a 1% aqueous solution of Intralipid using our system. Error bars represents the variability across all the detectors. (b) Reduced scattering spectrum of a 1% aqueous solution of Intralipid using our system. Error bars represents the variability across all the detectors.

We processed the data offline using Matlab R2015a. We used the classical method to estimate the reduced scattering coefficient ${\mu ^\prime
       _{\rm{s}}}$ and of the absorption coefficient ${\mu _{\rm{a}}}$, where the TPSFs are fitted using a standard model of diffusion theory [53], after convolution with the IRF. Fig. 8 reports the retrieved absorption and scattering coefficient. The error bars reflect the standard deviation across all the detectors. We see that we retrieve an absorption spectra in accordance with the literature [54], [55], with the classical shape of the water spectrum, and an absolute value in reasonable accordance with the previously reported value of the water absorption coefficient. For example, at 800 nm the absorption coefficient of the solution is ${\text{0.026}}\,{\text{cm}}^{-
      1}$. This is in reasonable accordance with the value of ${\text{0.022}}\,{\text{cm}}^{-
      1}$ reported by Kou and colleagues [54]. The deviation of less than 20% is in the same range as previously reported values in the literature for similar phantoms [56]. This is likely to be due to the presence of Intralipid which adds a small absorptivity [57], the purity of water and the temperature of the solution [58]. Regarding the reduced scattering coefficient, we note that we retrieve its classical spectral shape, with a decrease from ${\text{13.53}}\,{\text{cm}}^{-
      1}$ to ${\text{9.59}}\,{\text{cm}}^{- 1}$ for increasing wavelength. The absolute values are also in reasonable accordance with the literature. For example, at 830 nm the reduced scattering coefficient of the solution is ${\text{10.38}}\,{\text{cm}}^{- 1}$ . At the same Intralipid concentration, the value reported in [52] is ${\text{9.35}}\,{\text{cm}}^{-
       1}$. The deviation is of 11%, which is in the range of previously reported values [59] .

### C. nEUROPt

The nEUROPt protocol has been developed to assess and compare time resolved brain imaging systems. A complete description of the protocol can be found in Wabnitz and colleagues [44]. This protocol intends to “address the characteristic[s] of optical brain imaging to detect, localize, and quantify absorption changes in the brain”.

The data analysis was performed by dividing the reflectance curve in time gates with fixed width (500 ps) and variable delay from 0 ps (MTOF of the IRF), in 500 ps steps. The protocol was implemented with liquid phantoms based on calibrated Intralipid and ink [60] with black inclusions [61] (PVC cylinders of different sizes), mimicking a localized absorption perturbation at a certain depth. The protocol estimates the contrast, defined as:

\begin{equation*} {\rm{C}}({\rm{d}},{\rm{w}};{\rm{T}};\lambda) = - ({\rm{N}}({\rm{d}},{\rm{w}};{\rm{T}};\lambda) - {{\rm{N}}_0}({\rm{d}},{\rm{w}};\lambda))/{{\rm{N}}_0}({\rm{d}},{\rm{w}};\lambda),\tag{1} \end{equation*}

where N(d,w;T;λ) is the number of photons collected in a time window with delay, d, and width, w, at macroscopic (experiment) time, T, for wavelength, λ, and ${\text{N}}_{0}{{(\text{d},\text{w};\lambda)}}$ is the number of photons collected in the same time window and for the same wavelength, averaged over the baseline period of the protocol. In this experiment, a ${\text{100 mm}}^{3}$ PVC black cylinder was placed in the mid plane between source and detector (source detector distance 20 mm) inside the liquid (contained in the same cell used in the previous section) and moved in order to change its distance from the source-detector plane. In this way, it is possible to mimic a typical change in the absorption coefficient at different depths inside the liquid phantom. The measurements were repeated one hundred times.

**Fig. 9.**
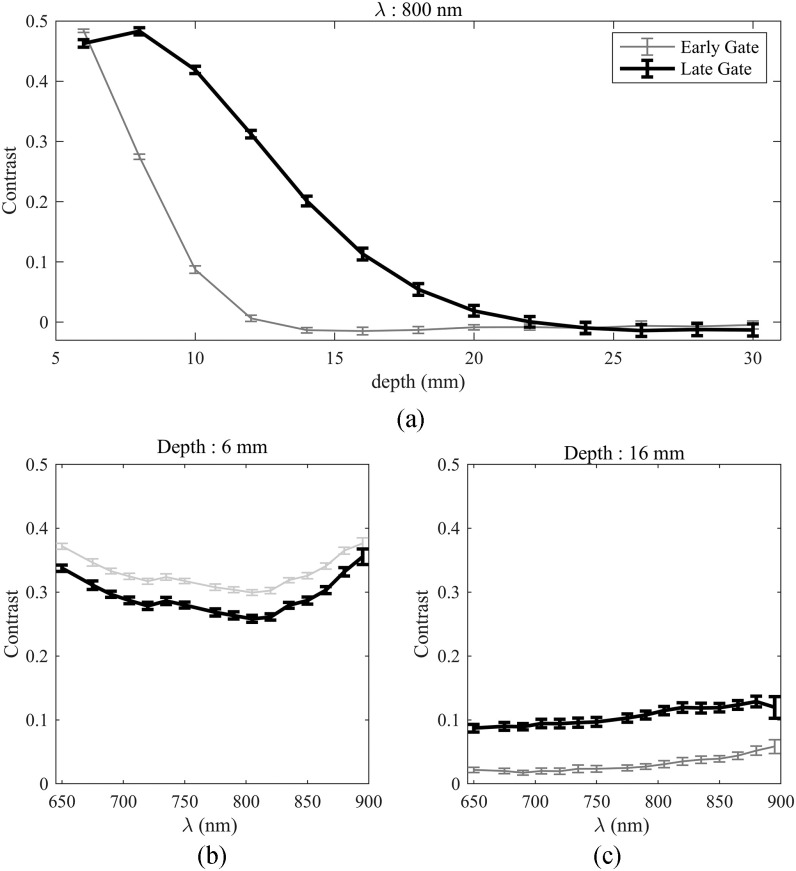
(a) Contrast at 800 nm for detector 4, for a target of ${\text{100 mm}}^{3}$, at depth between 6 mm and 30 mm, in steps of 2 mm. (b) Contrast, for detector 4, for a target of ${\text{100 mm}}^{3}$, at 6 mm of depth for 2 gates (early and late) for wavelengths between 650 and 900 nm in steps of 15 mm. (c) Contrast, for detector 4, for a target of ${\text{100 mm}}^{3}$, at 16 mm of depth for 2 gates (early and late) for wavelengths between 650 and 900 nm in steps of 15 mm. Error bars represent noise as obtained from the standard deviation of 100 repeated measurements.

We have evaluated the contrast at 800 nm for detector 4, for a target localized from 6 mm to 30 mm, in steps of 2 mm. Fig. 9(a) shows the contrast versus the target depth for 2 different time gates, one for early photons (time delay = 0), and one for late photons (Time delay = 3000 ps). For the early gate the contrast is high at small depths and decreases rapidly after 10 mm. The late gate trend is noticeably different, with contrast peaking at 8 mm and decreasing rapidly after 16 mm. Furthermore, we see that the contrast is still greater than 0.1 at 16 mm, and that the contrast is detectable until 22 mm.

We also tested the ability to detect that change at multiple wavelength across the full bandwidth of the system. Fig. 9(b) and (c). report the contrast for the same 2 gates as in Fig. 9(a), at 6 and 16 mm of depth, for wavelength between 650 nm and 895 nm, in steps of 15 nm. We retrieve the same results as in the 800 nm experiment, with a decrease in contrast when depth increases, for all wavelengths. We also note that the contrast of the late gate is greater than the one of the early gate at 16 mm, for all wavelengths. We see that the contrast is dependent on wavelength. This is explained by the fact that the target creates a contrast depending on the optical properties of the liquid surrounding it [62]. We can especially notice the 2 bumps around 730 and 800 nm of the absorption spectra of water, for the early gate at 6 mm. Finally, even though the responsivity of the detector falls after 850 nm, which is noticeable with the increase of the noise, notably for the late gate, we see that the contrast is still detectable up to 895 nm.

### D. Blood/Yeast Liquid Phantom

In order to validate the ability of the system to measure variations in the haemoglobin and oxCCO concentrations, we used homogeneous liquid phantoms based on blood and yeast. Blood phantoms have been used on several occasions to validate the ability of NIRS systems to retrieve absolute oxygenation information  [63], [64]. The basic principle of these phantoms is to measure [HHb] and [HbO_2_] over cycles of oxygenation and deoxygenation of the blood. Then total haemoglobin ([HBT] = [HHb]+ [HbO_2_]) is calculated to infer the tissue saturation level (StO_2_ =[HbO_2_]/[HBT]). The tissue saturation level can be changed from 100% to 0%. Typically, the blood is oxygenated by bubbling O_2_ gas through the liquid. Once the blood is fully oxygenated, the deoxygenation can be performed in two ways, either using yeast or nitrogen gas (${\text{N}}_{2}$). The ${\text{N}}_{2}$ method is relatively slow to use and is sensitive to the gas exchange between the phantom and the ambient air. The yeast method is simpler to use and deoxygenates the blood faster. Moreover, the yeast consumes the ${\text{O}}_{2}$ via the same process of aerobic respiration as in human tissue. Thus, the electron transport chain is involved and [oxCCO] can be detected. However, to our knowledge, [oxCCO] has never been investigated in this type of phantom. Thus, in order to validate the capability of our system to resolved [oxCCO], we have constructed 2 phantoms, one based on yeast, and one based on N_2_ bubbling for the deoxygenation. Therefore, we hypothesise that we will be able to detect the [oxCCO] change concomitant with the deoxygenation of the blood for the yeast phantom. Then, the second phantom based on ${\text{N}}_{2}$ will help to validate the fact that the [oxCCO] change is not due to crosstalk, and we hypothesise that there will be no significant changes in [oxCCO] in that phantom.

#### 1) Phantom Description and Setup

The phantom container consists of a metallic box (27 × 15 × 16 cm). The inner box was covered in black absorbing material to satisfy the semi-infinite boundary approximation. The container was filled with a Phosphate-buffered saline solution (PBS, P3813, Sigma-Aldrich, Germany) and Intralipid 20% (Fresenius Kabi Italia, Italy). The PBS is used to maintain the pH close to physiological values (i.e pH = 7.4) and the Intralipid is commonly used to introduce scattered centres in order to match the scattering properties of tissues [60], [65]. It worth mentioning that the quantities of the basal solution were weighed as it is easier to weigh liquids when dealing with large volumes. Moreover, it is a well-established way to measure volumes [57]. Then 15 mL of blood was added to the mixture. Here, a syringe was used as it was simple to extract the blood from the blood bag with it. Finally, the container was positioned on a hot stirring plate in order to keep both the temperature and homogeneity of the phantom constant during the whole measurement. The temperature of the solution was monitored and kept to 37 °C(+/−1 °C), and the solution was stirred throughout the whole experiment. Table I details the phantom's composition, where phantom 1 used yeast for the deoxygenation, and phantom 2 used ${\text{N}}_{2}$.

**TABLE I:** Phantom Compositions

*solution*				
Mixture	PBS (g)	Intralipid (g)	Blood (mL)	Deoxygenation
*N*°*1*	1435	75	15	Yeast (2 g)
*N*°*2*	1435	75	15	N2

**TABLE II:** Measurement Protocol

Step	Phantom 1	Phantom 2
1	Basal solution	
2	Add 2 g of yeast	Turn on N2
3	Wait for plateau	
4	Turn on O2	Turn off N2/Turn on O2
5	Wait for plateau	
6	Turn OFF O2	
7	Repeat step 3 to 6	
8	STOP	

For the gas supply, we used an industrial oxygen tank connected to an air stone positioned at the bottom of the phantom. The same was done for the nitrogen gas in phantom 2.

Both experiments used a single source and detector. The inter-optode distance was set to 3 cm and an optode holder was 3D printed specifically for this experiment. It was made of black material in order to satisfy the semi-infinite boundary approximation. The fibres were slightly submerged to avoid problems due to motion or bubbles at the top of the liquid.

As well as the TR system, we used a broadband CW NIRS system as a reference, since this technique is the gold standard for the NIRS measurement of [oxCCO] [10]. We used a modified version of system named CYRIL [11]. The instrument is based on a halogen light source for the illumination and a lens-based spectrograph for the detection. The illumination and detection stages are connected to the sample via optical fibres and for this experiment, we used a single source and detector.

**Fig. 10.**
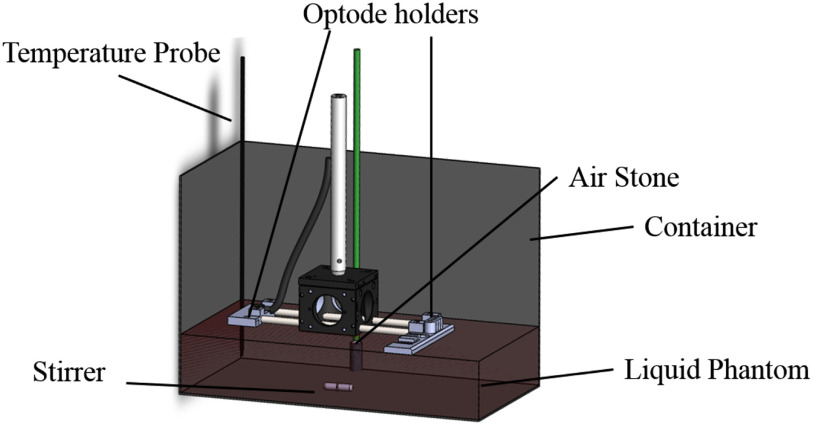
Schematic of the phantom measurement configuration. Optical fibres are not shown for clarity.

After the probes were positioned, a plastic film was positioned on top of the container, minimising the gas exchange between the ambient air and the phantom. Fig. 10 presents a 3D rendering of the experimental set-up.

#### 2) Measurement Protocol

For the TR measurement, an IRF was acquired before and after each phantom experiment. It worth noting that no significant difference was found in the IRF before and after the measurement, confirming the stability of the system. For these experiments we used 16 wavelengths (780, 786, 792 798, 804, 810, 816, 822, 828, 834, 840, 846, 852, 858 864, 870 nm). The integration time for each wavelength was set to 100 ms (MT = 20 ms × 5 repeats), resulting in an acquisition frequency of about 0.5 Hz. The measurement started after the temperature stabilized. The solution was measured for 5 mins before inducing the first deoxygenation to quantify the baseline conditions. After 5 mins, deoxygenation was induced either by adding 2 g of yeast or turning on the N_2_. Then, we waited for the plateau of the deoxygenation before turning on the O_2_. Finally, we waited for the oxygenation's plateau before turning off oxygen again and starting a new deoxygenation cycle.

#### 3) Data Processing

All data processing was performed offline using Matlab R2015a. Regarding the TR data, in order to estimate the reduced scattering coefficient ${\mu ^\prime _{\rm{s}}}$ and the absorption coefficient ${\mu _{\rm{a}}}$ the TPSFs were fitted using a standard model of diffusion theory  [53], after convolution with the IRF. We then calculated the absolute concentration of [HHb], [HbO_2_] and [oxCCO], by assuming a water concentration of 98%. The [HHb], [HbO_2_] data were then used to calculate the absolute saturation of the phantom.

In order to compare the TR and CW results, we finally calculate the change in concentrations Δ[HHb], Δ[HbO_2_] and Δ[oxCCO], using the modified Beer Lambert law[13], based on the $\Delta {\mu _{\rm{a}}}$ defined as: $\Delta {\mu _a}(T) = {\mu _{a0}} - {\mu
       _a}(T)$, with ${\mu
        _{a0}}$ being the mean value during the first 5 mins of the experiment and $\mu _{{\text{a}}}{\text{(T)}}$ being the ${\mu}
        _{{\text{a}}}$ value at time T.

Regarding the CW data, we used the UCLn algorithm [13] to calculate the change in concentrations Δ[HHb], Δ[HbO_2_] and Δ[oxCCO]. The wavelength range used for the calculation was 770 to 900 nm. We used the method described in Matcher and colleagues to calculate the pathlength [66] used in the UCLn algorithm, in order to extract absolute changes in concentrations and compare it to the TR calculation. It worth noting that we used the extinction coefficient available on the UCL website (http://www.ucl.ac.uk/medphys/research/borl/intro/spectra). 

#### 4) Results

**Fig. 11.**
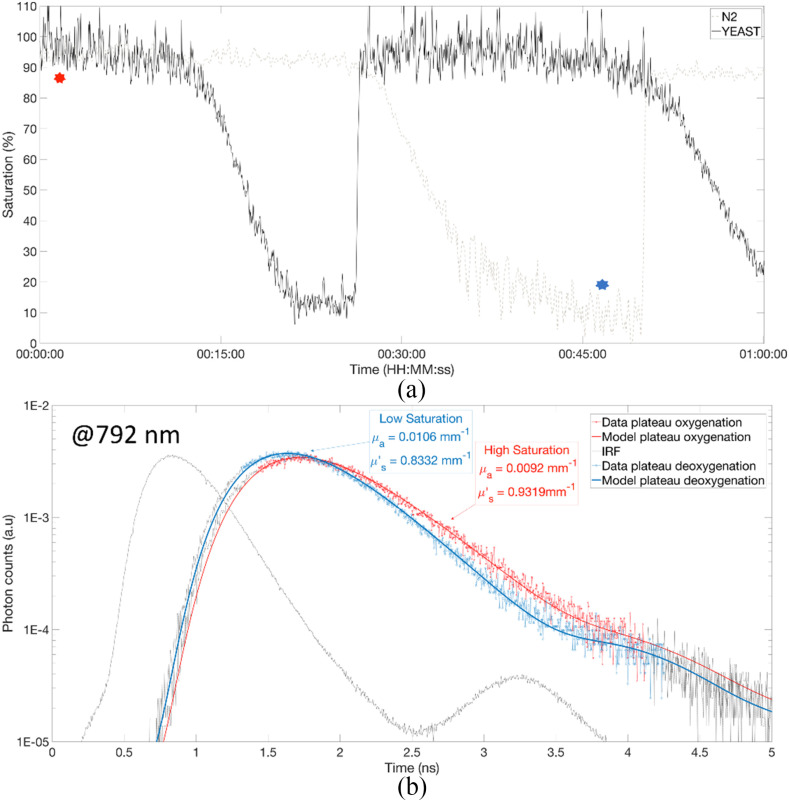
(a) evolution of the saturation of the yeast (solid black) and ${\text{N}}_{2}$ (dashed grey) phantoms over the first hour of the experiment. (b) Example of the fitting of the optical properties for the ${\text{N}}_{2}$ phantom at 792 nm. The blue curve represents a low saturation point and the red curve represents a high saturation point. The exact timing of those points is reported with stars on part (a).

Fig. 11(a) shows the saturation level during the first hour of the experiment for the ${\text{N}}_{2}$ and yeast phantom. As expected, the starting values are very close to 100% for both phantoms. We note a different time dynamic between the ${\text{N}}_{2}$ and yeast phantom. Indeed, the ${\text{N}}_{2}$ phantoms requires about twice the time (40 min) of the yeast phantom (20 min) to reach the deoxygenated plateau. For this reason, we will only report the first cycle for that phantom in the comparison between the absolute change in chromophores. Finally, we see that the deoxygenation plateau for ${\text{N}}_{2}$ is very close to 0%, whereas the one of the yeast is of about 10%, which is in the range of previously developed phantoms [64]. This might be due to the low quantity of yeast used, which perhaps wasn't enough to fully deoxygenate the phantom.

**Fig. 12.**
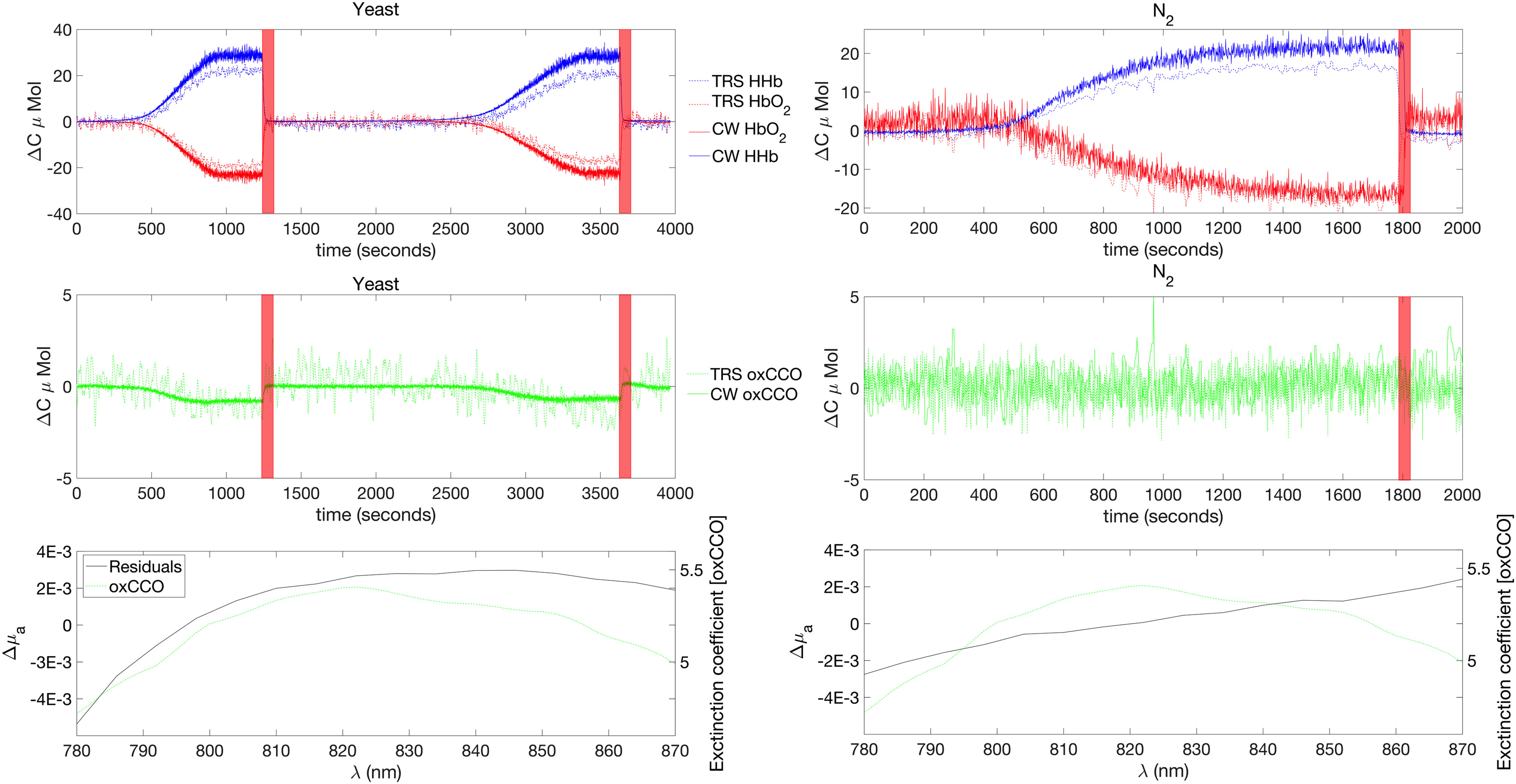
Summary of the phantom results. From top to bottom. Concentration changes in [HHb] and [HbO_2_] for both the TR and CW instrument, Concentration changes in [oxCCO] for both the TR and CW instrument, Residuals of the 2 and 3 components fit together with the extinction coefficient of the [oxCCO]. Left and right columns refer to yeast and ${\text{N}}_{2}$ phantom respectively. The red shaded regions correspond to the O_2_ ON period.

Fig. 11(b) reports an example of the fitting of the absorption and reduced scattering properties of the phantoms. It shows the IRF and the TPSF at 792 nm, for the ${\text{N}}_{2}$ phantom, both at the maximum and minimum saturation plateau. The time of those two events are marked with a blue (deoxygenation plateau) and a red star (oxygenation plateau) on Fig. 11(a). We retrieved the expected increase in absorption coefficient with the desaturation at 792 nm (i.e wavelength sensitive to deoxyhaemoglobin), from ${\text{0.0092}}\,{\text{mm}}^{-
       1}$ to ${\text{0.0106}}\,{\text{mm}}^{- 1}$. Fig. 12 reports the results of the two phantom measurements. The left and right columns report the results of the yeast and ${\text{N}}_{2}$ phantoms respectively. The first line reports the changes in [HHb] and [HbO_2_] for both instruments, and the second line reports the changes in [oxCCO] for both instruments. Finally, the last line reports the residuals analysis compared to the extinction coefficient of [oxCCO]. The residual analysis enables an observation of the accuracy of the fit of the chromophore spectra to the measured attenuation  [10], [11], [67], [68]. Thus, we have re-calculated the concentration changes with only [HHb] and [HbO_2_]. Then the absorption change spectra were back-calculated from these concentration changes and the differences between the 2- and 3- chromophore fits were studied.

Regarding the concentration changes, we see that the results are very similar between the two instruments. For the yeast phantom, the 2 cycles are similar in terms of values and dynamic. We note an increase in [HHb] together with a decrease in [HbO_2_] and [oxCCO]. For the ${\text{N}}_{2}$ phantom, [HHb] and [HbO_2_] are similar to the yeast phantom. In terms of [oxCCO], we see that there is no significant variation during the course of the experiment.

Finally, the residual analysis demonstrates that the changes in [oxCCO] retrieved in the yeast phantom are not due to crosstalk. Indeed, the residual of the 2-3 chromophore fit is very similar to the [oxCCO] spectra in that phantom. However, for the ${\text{N}}_{2}$ phantom, the residual shape is rather flat, and does not correspond to the [oxCCO] spectrum.

The comparison between the yeast and ${\text{N}}_{2}$ phantoms validates the capability of the system to detect [oxCCO]. Indeed, by taking the N_2_ phantom as a reference for invariant [oxCCO], we can confirm that the [oxCCO] variation detected in phantom 1 is not due to crosstalk.

## IV. Discussion

We have described a multi-wavelength, multichannel time resolved system that is able to retrieve both the haemoglobin and oxCCO concentration changes. We used well defined protocols to characterise the system. The BIP protocol confirms the fact that the basic system components behave like previously developed TR systems, in terms of output power, responsivity, IRF characteristic, and warm up time. The estimation of the optical properties of a 1% Intralipid aqueous solution confirm the ability of the system to retrieve accurately the optical properties of a turbid medium. Lastly, the nEUROPt protocol confirms the capability of the system to detect changes in absorption comparable to absorption changes encountered during typical brain activation. Indeed, we used a target of ${\text{100
      mm}}^{3}$ that mimics typical brain activation [61]. Moreover, it appears that the contrast is detectable over the whole bandwidth of the system. However, the integration time was set to 1s for every wavelength, as specified by the protocol. Depending on the application, and the time constraint of the acquisition, the useable bandwidth might be reduced. Thus, the wavelengths used for every application will have to be determined carefully.

We have also used a homogeneous phantom based on blood and yeast to assess the ability of the system to resolve [oxCCO] in the presence of large concentrations of haemoglobin. These phantoms allow to compare instruments [64], and test different parameters of the system. We are currently using these phantoms to investigate the effect of the wavelength selection on the calculated concentration. We are also testing the number and the position in the spectra of every wavelength to enhance the capabilities of the system in terms of acquisition frequency and accuracy of the calculated concentration. Moreover, we are working on the precise calibration of those phantoms in terms of haemoglobin and oxCCO concentrations. Indeed, our ultimate goal is to be able to retrieve an absolute quantification of the metabolic state of tissue. Thus, we will have to precisely calibrate our phantom in order to know its absolute oxidise and reduced CCO concentration.

**Fig. 13.**
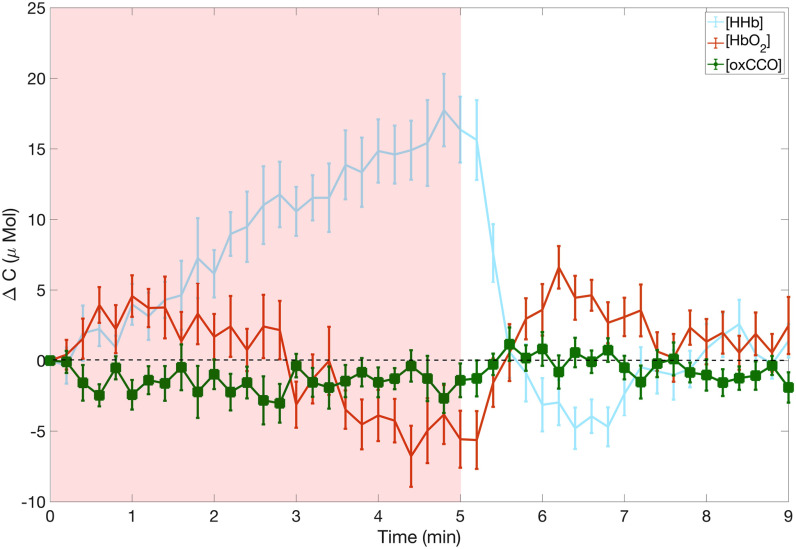
Concentration changes for [HHb], [HbO2] and [oxCCO] consecutive to a muscular cuff occlusion. Reproduced from reference [42].

Finally, we stress that we report here only the phantom results. We previously reported our first *in-vivo * validation [42] where we have reported the changes in oxygenation and metabolism in the left forearm of 5 healthy volunteers during a 5 min occlusion of the brachial artery. Fig. 13 shows the grand average over all the subjects of the temporal evolution of the concentration changes in [HHb], [HbO_2_] and [oxCCO] induced by the occlusion. We retrieved the classical haemodynamic responses to this type of challenge, with an increase in [HHb] and a decrease in [HbO_2 _], and a maximum change occurring at the end of occlusion. The [oxCCO] shows a different dynamic response compared with the haemoglobins. Matcher and colleagues postulated that the changes in [oxCCO] during muscle ischaemia should be small [13]. This behaviour was observed in our study and our results are in good agreement with the literature [13]. The small change of [oxCCO] was a good indication of the absence of crosstalk between haemoglobin and oxCCO, which is one of the major issues when trying to resolve the [oxCCO] [10]. Indeed, in the presence of crosstalk, the changes in magnitude of the [oxCCO] during a forearm ischemia can be the same as that of the haemoglobins [13].

Thus, the phantom study, which mimics ischemia, is in good agreement with our first *in-vivo* study in terms of temporal dynamic and in terms of concentration changes. As one of the primary interests of our group is to follow ischemia in neonates [11], this first study is encouraging in order to move to further *in-vivo* studies. Moreover, the first nEUROPt protocol results presented here indicate that the system should be suitable to monitor the brain during a functional study in adults; which is what we are currently exploring.

## V. Conclusion

We have developed a multi-wavelength multichannel TR system that is able to retrieve absorption and scattering changes and quantify absolute concentrations of haemoglobin and oxCCO concentration changes. We have assessed the basic behaviour of the system on well-established phantoms and validated its ability to resolve haemoglobin and oxCCO in calibrated phantoms. We plan to extend our work on the phantom to optimize the parameters of the system (such as number and selection of wavelengths); following which we will perform brain tissue *in-vivo* studies. 
